# Non-thyroidal illness syndrome subtypes and mortality in sepsis: associations with thyroid autoantibodies

**DOI:** 10.3389/fendo.2026.1767422

**Published:** 2026-03-27

**Authors:** Yi Zhou, Xiangtao Zheng, Yanjun Zheng, Zhitao Yang

**Affiliations:** Department of Emergency Medicine, Ruijin Hospital, Shanghai Jiao Tong University School of Medicine, Shanghai, China

**Keywords:** in-hospital mortality, non-thyroidal illness syndrome, sepsis, thyroid autoantibodies, thyroid hormones

## Abstract

**Background:**

Non-thyroidal illness syndrome (NTIS) is frequently observed in patients with sepsis; however, prognostic differences among its subtypes remain poorly defined. This study aimed to evaluate whether different NTIS subtypes and the presence of thyroid autoantibodies are associated with mortality in septic patients.

**Methods:**

We conducted a retrospective cohort study including 871 patients with sepsis admitted to a tertiary hospital in Shanghai between 2015 and 2019. Thyroid function tests were performed within 24 hours of sepsis diagnosis. NTIS was categorized into four subtypes according to serum free triiodothyronine (FT3), free thyroxine (FT4), and thyroid-stimulating hormone (TSH) levels: NTIS-1 (isolated low T3), NTIS-2 (combined low T3 and T4), NTIS-3 (low T3 with preserved TSH response), and NTIS-4 (near-normal thyroid hormone levels with mild abnormalities). Thyroid antibodies, including thyroglobulin antibody (TGAb) and thyroid peroxidase antibody (TPOAb), as well as other clinical and laboratory parameters, were measured. The primary outcome was in-hospital mortality. Multivariable logistic regression analyses were performed, adjusting for age, sex, comorbidities, Sequential Organ Failure Assessment (SOFA) score, infection site, and relevant laboratory variables.

**Results:**

NTIS was present in 82% of patients (715/871). Compared with patients without NTIS, those with NTIS were older, had a higher burden of comorbidities, and exhibited more severe organ dysfunction, as reflected by higher SOFA scores (all p < 0.01). After multivariable adjustment, NTIS remained an independent predictor of in-hospital mortality, conferring more than a threefold increased risk (odds ratio [OR] 3.14, 95% confidence interval [CI] 1.52–7.00, p = 0.003). Among NTIS subtypes, both NTIS-1 (OR 4.26, 95% CI 1.33–16.41, p = 0.022) and NTIS-2 (OR 3.99, 95% CI 1.31–14.88, p = 0.024) were associated with approximately a fourfold higher risk of death. TGAb positivity emerged as the strongest prognostic factor, doubling the risk of mortality in the overall cohort (OR 2.15, 95% CI 1.30–3.60, p < 0.01) as well as in the NTIS subgroup (OR 1.97, 95% CI 1.17–3.33, p = 0.011). TPOAb positivity showed a similar trend toward increased mortality but did not reach statistical significance (OR 1.74, 95% CI 0.96–3.14, p = 0.068).

**Conclusions:**

NTIS is highly prevalent among patients with sepsis and independently predicts in-hospital mortality after adjustment for disease severity. Patients with NTIS-1 and NTIS-2 subtypes are at particularly high risk of death. Moreover, TGAb positivity is a strong and independent predictor of mortality. Incorporating thyroid function tests together with thyroid antibody measurements may enhance risk stratification in patients with sepsis.

## Introduction

Sepsis remains one of the leading causes of death among hospitalized patients worldwide (1, 2). Despite substantial advances in intensive care management and antimicrobial therapy, mortality remains unacceptably high, ranging from 15–30% in sepsis and exceeding 40% in septic shock (3). The profound systemic inflammatory and metabolic stress associated with sepsis disrupts multiple endocrine pathways, including the hypothalamic–pituitary–thyroid (HPT) axis (4, 5).

Non-thyroidal illness syndrome (NTIS) is highly prevalent in critically ill patients and is typically characterized by reduced serum triiodothyronine (T3) levels accompanied by normal or decreased thyroxine (T4) and thyroid-stimulating hormone (TSH) concentrations (6, 7). In intensive care unit (ICU) settings, particularly among patients with sepsis or septic shock, the prevalence of NTIS can reach 70–80% (8, 9). Pathophysiologically,NTIS reflects disturbances in peripheral thyroid hormone metabolism, including impaired T4-to-T3 conversion, increased production of reverse T3 (rT3), and dysregulation of hypothalamic–pituitary feedback mechanisms (10, 11). Whether these alterations represent an adaptive response to reduce energy expenditure or a maladaptive process that contributes to organ dysfunction remains a subject of ongoing debate (12, 13).

Findings from multiple observational studies and meta-analyses indicate that NTIS is linked to increased mortality in sepsis and other critically ill populations (5, 14, 15). Reduced T3 levels have been correlated with greater disease severity, prolonged hospitalization, and a higher risk of death (16, 17). However, most existing studies have treated NTIS as a homogeneous condition, thereby overlooking potential heterogeneity in thyroid hormone profiles (6, 9). Distinct NTIS subtypes—such as isolated low T3, combined low T3 and T4, or abnormal TSH responses—may reflect different underlying pathophysiological mechanisms and carry different prognostic implications, yet these distinctions remain insufficiently explored (16, 19).

In addition to alterations in thyroid hormone levels, thyroid autoimmunity may further modulate outcomes in sepsis. Thyroglobulin antibodies (TGAb) and thyroid peroxidase antibodies (TPOAb) are relatively common in the general population, with approximately 10–15% of adults testing positive (20). Emerging evidence suggests that autoimmune thyroid antibodies may interfere with innate immune function and cytokine regulation, potentially influencing the host response to infection and the clinical course of sepsis (21, 22). Experimental studies have also shown that thyroid hormones can enhance nitric oxide–mediated bacterial clearance and improve survival in infectious models (23). Nevertheless, clinical data evaluating the prognostic significance of thyroid antibodies in septic patients, particularly in the context of NTIS, remain scarce (24).

To address these knowledge gaps, we conducted the present study with three specific objectives. First, we assessed the prevalence of NTIS and its subtypes in patients with sepsis, classifying NTIS into four subtypes based on patterns of free triiodothyronine (FT3), free thyroxine (FT4), and TSH. Second, we examined whether different NTIS subtypes are associated with differential risks of mortality. Third, we evaluated whether the presence of thyroid autoantibodies provides prognostic information beyond established clinical severity scores. We hypothesized that integrating NTIS subtyping with thyroid antibody assessment would improve risk stratification in patients with sepsis.

## Materials and methods

### Study design and data source

We conducted a single-center retrospective cohort study using data from a tertiary teaching hospital in Shanghai, China. The study period spanned from January 1, 2015, to December 31, 2019. Data were obtained from the hospital information system, including electronic medical records, the laboratory information system, and the computerized order entry system. Structured queries were used to extract relevant variables. The study protocol was approved by the Ethics Committee of Ruijin Hospital, Shanghai Jiao Tong University School of Medicine (approval number: 2021-59; March 9, 2021). Because all data were de-identified and analyzed retrospectively, the requirement for informed consent was waived.

### Study population

All adult inpatients (aged ≥18 years) with a discharge diagnosis of sepsis during the study period were screened. The date of the first sepsis diagnosis during the index hospitalization was defined as the baseline date. This initial screening identified 1,860 potential sepsis cases.

Patients were eligible for inclusion if they met all of the following criteria: (1) age ≥18 years; (2) hospitalization with a discharge diagnosis of sepsis; and (3) availability of at least one complete thyroid function panel—free triiodothyronine (FT3), free thyroxine (FT4), and thyroid-stimulating hormone (TSH)—measured within 24 hours after the sepsis diagnosis.

Exclusion criteria were applied sequentially as follows: (1) absence of thyroid function testing during the septic episode (n = 899); (2) missing key clinical information required to assess sepsis severity or infection characteristics, including components of the Sequential Organ Failure Assessment (SOFA) score, Systemic Inflammatory Response Syndrome (SIRS) criteria, or infection site; (3) use of systemic glucocorticoids before the first thyroid panel during the septic episode; and (4) pre-existing thyroid disease or prior thyroid surgery that could substantially alter baseline thyroid function. The latter category included hyperthyroidism, hypothyroidism, Hashimoto thyroiditis with abnormal thyroid function, total thyroidectomy, and partial thyroidectomy requiring long-term hormone replacement.

After applying all inclusion and exclusion criteria, 871 patients constituted the final study cohort. Among them, 715 patients met the criteria for non-thyroidal illness syndrome (NTIS), while 156 patients were classified as euthyroid according to the definitions described below. Each patient contributed only one index hospitalization and one set of baseline thyroid measurements.

### Thyroid status and biomarker definitions

#### Thyroid status and NTIS subtypes

Thyroid function was evaluated using the first measurements of FT3, FT4, and TSH obtained within 24 hours after sepsis diagnosis. The reference ranges used at our institution were 2.63–5.70 pmol/L for FT3, 9.01–19.04 pmol/L for FT4, and 0.35–4.94 μIU/mL for TSH. In addition, total triiodothyronine (T3; reference range 0.89–2.44 nmol/L) and total thyroxine (T4; reference range 62.67–150.84 nmol/L) were measured.

Euthyroid status was defined as FT3, FT4, and TSH values all within their respective reference ranges. NTIS was defined by an FT3 concentration below the lower limit of normal, with or without concomitant abnormalities in FT4 or TSH. Patients with NTIS were further categorized into four subtypes based on predefined hormonal patterns: NTIS-1 (low FT3 with normal FT4 and normal TSH), NTIS-2 (low FT3 and low FT4 with normal or low TSH), NTIS-3 (low FT3 with elevated TSH above the upper reference limit), and NTIS-4 (other thyroid dysfunction patterns not meeting the criteria for NTIS-1 to NTIS-3). This classification followed a prespecified algorithm illustrated in the study flowchart.

#### Hormone and antibody assays

Serum TSH, FT3, FT4, total T4 (TT4), total T3 (TT3), and reverse T3 (rT3) were measured by automated chemiluminescent immunoassays using the Architect i2000SR platform (Abbott Laboratories, Chicago, IL). The laboratory reference ranges provided by the manufacturer were applied in this study.

Serum thyrotropin receptor antibody (TRAb) levels were determined using electro-chemiluminescence immunoassays (Cobas 601 analyzer; Roche Diagnostics), with a recommended cutoff value of 1.75 IU/L.

Serum thyroid peroxidase antibodies (TPOAb) and thyroglobulin antibodies (TgAb) were measured using chemiluminescent microparticle immunoassays (Abbott Laboratories, Abbott Park, IL). Serum thyroglobulin concentrations were determined by immunometric assay (Cobas e601; Roche Diagnostics) in the same certified central laboratory.

All assays were performed in accordance with the manufacturers’ protocols in a certified clinical laboratory.

#### Thyroid-related biomarkers

Four additional thyroid-related biomarkers measured within the same time window as the thyroid function panel were analyzed. Using local laboratory reference cutoffs, these markers were dichotomized as follows: thyroid peroxidase antibody (TPOAb) positivity (≥5.61 IU/mL), thyroglobulin antibody (TGAb) positivity (≥4.11 IU/mL), elevated reverse T3 (rT3 >95 ng/dL), and elevated thyroglobulin (>77 ng/mL). These biomarkers were used to compare prevalence across euthyroid and NTIS phenotypes and to assess their associations with clinical outcomes.

### Clinical variables and outcomes

Clinical variables were extracted from the hospital information system using structured queries, with manual chart review performed when necessary to ensure data accuracy. Collected demographic variables included age and sex. Comorbidities were summarized using the Charlson comorbidity index, and specific conditions such as diabetes mellitus, history of deep venous thrombosis (DVT), smoking status, and alcohol use were recorded. Infection sites were categorized as abdominal, respiratory, urinary, bloodstream, central nervous system, skin or soft tissue, or unknown.

Baseline disease severity was assessed using the SOFA score, SIRS criteria count, quick SOFA (qSOFA) score, and Acute Physiology and Chronic Health Evaluation II (APACHE II) score. Vital signs around the time of sepsis diagnosis included body temperature, heart rate, mean arterial pressure, respiratory rate, oxygen saturation, and Glasgow Coma Scale score. Laboratory parameters were collected within a ±2-day window around the diagnosis of sepsis and included complete blood count, liver function tests, renal function indices, electrolytes, coagulation parameters, inflammatory markers, lactate, and cardiac troponin I. Treatment-related variables included the use of mechanical ventilation and vasopressors.

The primary outcome was in-hospital mortality, defined as death occurring during the index hospitalization. The secondary outcome was hospital length of stay, calculated in days from admission to discharge or death. Patients discharged alive were classified as survivors in the mortality analysis.

### Statistical analysis

All statistical analyses were performed using R software (version 4.2.3). Continuous variables were summarized as medians with interquartile ranges due to skewed distributions, while categorical variables were reported as counts and percentages.

#### Baseline comparisons

Baseline characteristics were compared between euthyroid and NTIS groups using the Mann-Whitney test for continuous variables and the chi-square test or Fisher’s exact test, as appropriate, for categorical variables.

#### Thyroid hormone and marker comparisons

Thyroid hormone levels (FT3, FT4, TSH, total T3, and total T4) and thyroid-related biomarkers (TPOAb, TGAb, rT3, and thyroglobulin) were compared across the euthyroid group and the four NTIS subtypes. Kruskal–Wallis tests were used for continuous variables, and chi-square tests were applied to categorical variables. To visualize differences in biomarker prevalence, a heatmap was generated showing positivity rates across the euthyroid, NTIS-1, and NTIS-2 groups. NTIS-3 and NTIS-4 were excluded from this visualization because of limited sample sizes (n = 5 and n = 57, respectively).

#### Association between NTIS and mortality

The association between thyroid status and in-hospital mortality was examined using binary logistic regression models. Unadjusted analyses assessed thyroid status as the sole predictor, followed by multivariable analyses adjusting for key demographic and clinical factors known to influence sepsis outcomes. Covariates included age, sex, Charlson comorbidity index, SOFA score, infection site, serum creatinine, D-dimer, total bilirubin, troponin I, white blood cell count, and neutrophil count.

In-hospital mortality served as the dependent variable, and thyroid status or NTIS subtype was the primary independent variable. Model performance and fit were evaluated to ensure the robustness of estimates. Regression results were reported as odds ratios (ORs) with 95% confidence intervals (CIs), and adjusted effects were visualized using forest plots.

#### Association between thyroid markers and outcomes

For each thyroid-related biomarker (TPOAb positivity, TGAb positivity, elevated rT3, and elevated thyroglobulin), analyses were conducted in both the overall sepsis cohort and the NTIS subgroup. Crude mortality rates were first compared between biomarker-positive and biomarker-negative patients using chi-square tests. Subsequently, multivariable logistic regression models were fitted for each biomarker, adjusting for the same covariates used in the NTIS analyses. Both crude and adjusted ORs with 95% CIs are reported.

Hospital length of stay was compared between biomarker-positive and biomarker-negative groups using the Mann-Whitney test in both populations. Results are presented as medians with interquartile ranges, and violin plots were used to illustrate the distribution of length of stay according to biomarker status.

#### General statistical principles

All analyses were performed using complete-case data; no multiple imputation was applied for missing values. All statistical tests were two-sided, and a p-value <0.05 was considered statistically significant.

All analyses and data visualizations were implemented using R software (version 4.2.3) with the following packages: *tidyverse*, *tableone*, *ggplot2*, *dplyr*, *forestplot*, *ggpubr*, and *reshape2*.

## Results

### Patient selection and baseline characteristics

**Figure 1:**
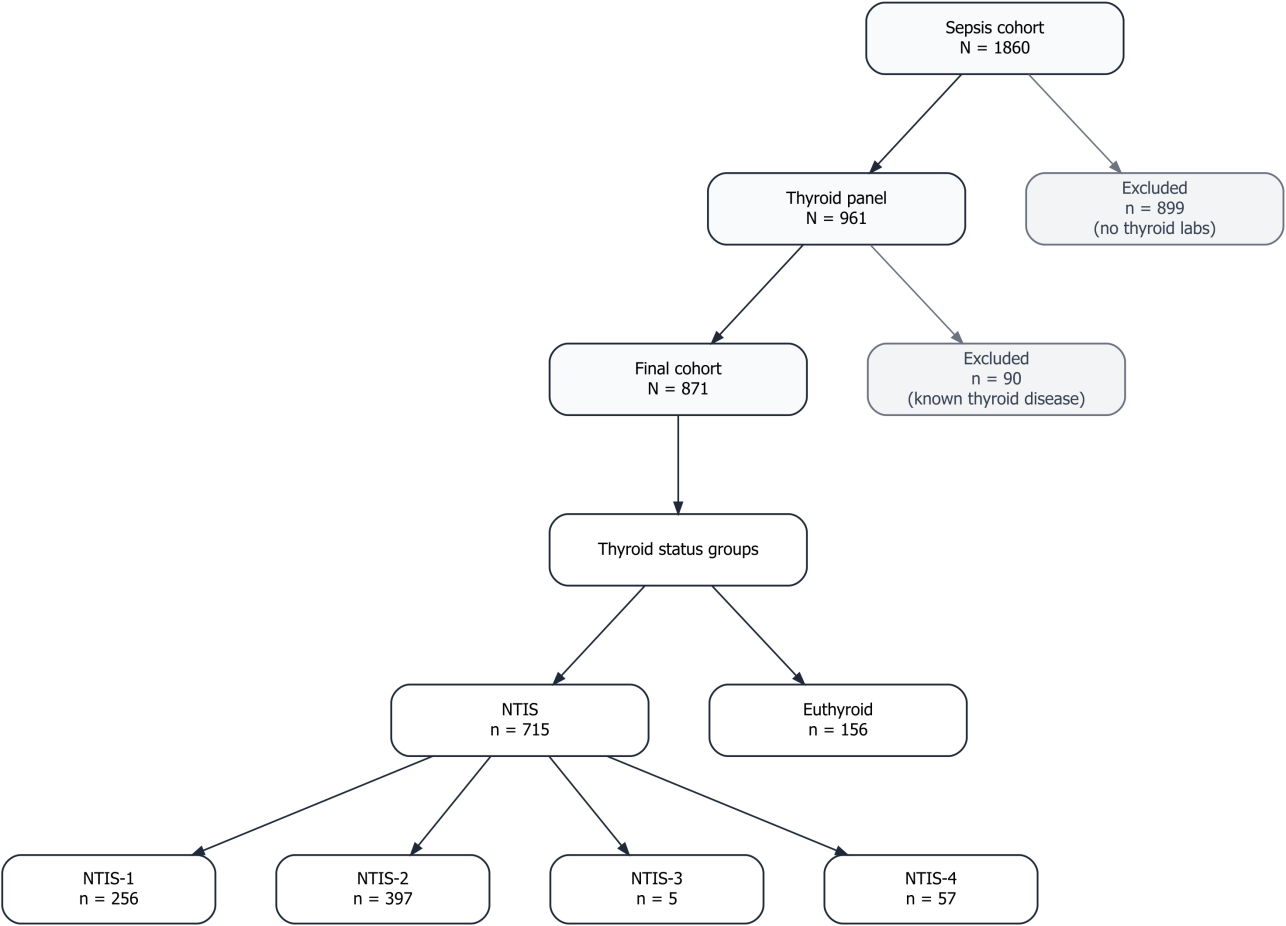
Study cohort selection and thyroid status classification. A total of 1,860 patients with sepsis were initially screened. After exclusion of patients without thyroid function testing (n = 899) and those with known thyroid disease, 871 patients were included in the final cohort. Patients were categorized into non-thyroidal illness syndrome (NTIS; n = 715) and euthyroid (n = 156) groups. The NTIS group was further subdivided into four predefined subtypes: NTIS-1 (n = 256), NTIS-2 (n = 397), NTIS-3 (n = 5), and NTIS-4 (n = 57).

Our final cohort included 871 sepsis patients: 715 with NTIS and 156 euthyroid (**Figure 1**). NTIS patients were distributed as follows: NTIS-1 (n=256), NTIS-2 (n=397), NTIS-3 (n=5), and NTIS-4 (n=57).

NTIS patients were older and sicker than euthyroid patients. More NTIS patients were over 65 years old (41.4% vs 29.5%, p<0.01). The median SOFA score was higher in NTIS (4 vs 1, p<0.01), indicating more severe organ dysfunction. Sex distribution was similar between groups (63% male in both, p=0.846). NTIS patients also had more comorbidities. Diabetes was twice as common in NTIS (20.0% vs 9.3%, p<0.01). DVT history was more frequent (38.9% vs 25.0%, p<0.01). The Charlson comorbidity index was higher in NTIS (p<0.01), reflecting their greater disease burden.

Vital signs showed that NTIS patients had more physiologic stress. Heart rate was faster (123 vs 114 per minute, p<0.01) and temperature was higher (39.0 vs 38.8 °C, p=0.040). Interestingly, respiratory rate was lower in NTIS (13 vs 14 per minute, p<0.01), which may reflect central drive suppression. Laboratory tests confirmed more widespread organ dysfunction in NTIS patients. They had higher white blood cell counts (9.4 vs 7.0 ×10 (9)/L, p<0.01) and neutrophil counts (7.6 vs 5.0 ×10 (9)/L, p<0.01), suggesting stronger inflammatory response. Kidney function was worse, with higher creatinine (78 vs 67 μmol/L, p<0.01). Markers of coagulation and tissue injury were also elevated: D-dimer was doubled (4.4 vs 2.2 μg/mL, p<0.01), bilirubin was higher (18.7 vs 15.4 μmol/L, p<0.01), and troponin I was elevated (0.07 vs 0.02 ng/mL, p<0.01). Respiratory infections were the most common source in NTIS patients (42.5% vs 29.7%) (**Table 1**).

**Table 1:** Baseline characteristics of patients with euthyroid and NTIS.

Variable	Euthyroid (N = 156)	NTIS (N = 715)	p value
Demographics			
Age, years			<0.01
18-39	43 (27.6)	117 (16.4)	
40-64	67 (42.9)	302 (42.2)	
>65	46 (29.5)	296 (41.4)	
Male sex	99 (63.5)	445 (62.2)	0.846
Vital signs			
Heart rate	114 (95-138)	123 (108-139)	<0.01
MAP, mm Hg	78 (72-86)	78 (71-89)	0.625
Respiratory rate	14 (12-16)	13 (11-15)	<0.01
Temperature, °C	38.8 (37.7-39.7)	39.0 (38.2-39.8)	0.040
GCS score	15 (15-15)	15 (15-15)	<0.01
Comorbidities and risk factors			
Diabetes mellitus	14 (9.3)	139 (20.0)	<0.01
History of DVT	39 (25.0)	278 (38.9)	<0.01
Current smoking	36 (23.1)	190 (26.6)	0.423
Alcohol use	20 (12.8)	147 (20.6)	0.035
Infection site			
Respiratory	44 (29.7)	44 (29.7)	
Abdominal	33 (22.3)	198 (28.6)	
Urinary	4 (2.7)	14 (2.0)	
Bloodstream	2 (1.4)	8 (1.2)	
Central nervous system	2 (1.4)	5 (0.7)	
Skin/soft tissue	5 (3.4)	6 (0.9)	
Unknown	58 (39.2)	167 (24.1)	
SOFA score	1 (0-5)	4 (1-7)	<0.01
Septic shock	10 (6.4)	77 (10.8)	0.134
Laboratory values			
White blood cell count, ×10^9^/L	7.0 (4.4-11.7)	9.4 (6.1-14.0)	<0.01
Neutrophil count, ×10^9^/L	5.0 (2.6-9.8)	7.6 (4.7-11.5)	<0.01
Hemoglobin, g/L	104 (82-124)	99 (79-117)	0.151
Hematocrit, %	31 (25-37)	30 (24-35)	0.099
Platelet count, ×10^9^/L	173 (95-271)	142 (73-229)	0.058
PT, sec	13.3 (12.3-15.1)	14.0 (12.7-15.9)	0.016
APTT, sec	33.1 (29.4-37.3)	33.5 (29.6-39.8)	0.263
D-dimer, μg/mL	2.2 (1.0-5.5)	4.4 (1.9-8.7)	<0.01
Fibrinogen, g/L	3.4 (2.4-4.9)	3.8 (2.5-5.1)	0.122
ALT, U/L	28 (16-51)	25 (13-52)	0.216
AST, U/L	28 (20-58)	33 (20-64)	0.245
Total bilirubin, μmol/L	15.4 (11.0-23.3)	18.7 (11.7-32.8)	<0.01
Creatinine, μmol/L	67 (51-84)	78 (56-130)	<0.01
Sodium, mmol/L	139 (136-142)	138 (135-143)	0.933
Potassium, mmol/L	3.9 (3.6-4.2)	3.8 (3.5-4.2)	0.715
Lactate, mmol/L	2.1 (1.7-2.9)	2.0 (1.5-2.9)	0.341
Arterial pH	7.42 (7.38-7.45)	7.42 (7.38-7.45)	0.757

Continuous variables: median (interquartile range); categorical variables: No. (%). Comparisons used Mann-Whitney tests (continuous) and chi-square or Fisher’s exact tests (categorical). MAP, mean arterial pressure; GCS, Glasgow Coma Scale; DVT, deep vein thrombosis; SOFA, Sequential Organ Failure Assessment; PT, prothrombin time; APTT, activated partial thromboplastin time; ALT, alanine aminotransferase; AST, aspartate aminotransferase; NTIS, non-thyroidal illness syndrome.

To evaluate potential selection bias related to thyroid function testing, baseline characteristics of patients included in the final cohort were compared with those who did not undergo early thyroid assessment. These comparisons are presented. (**Supplementary Table 1**). Although certain demographic and clinical differences were observed, overall severity indices and mortality rates were broadly comparable between groups, suggesting that the inclusion criteria did not introduce substantial imbalance in baseline disease severity.

### Thyroid hormone patterns

Euthyroid patients had median values within normal ranges for all thyroid hormones: FT3 3.58 pmol/L, FT4 14.16 pmol/L, TSH 1.74 μIU/mL, total T3 1.33 nmol/L, and total T4 101.16 nmol/L.

The four NTIS subtypes showed distinct hormone patterns (all comparisons p<0.01). NTIS-1 and NTIS-2 shared some features but differed in important ways. Both had low FT3 (2.33 and 1.81 pmol/L) and low total T3 (0.81 and 0.66 nmol/L). In both subtypes, TSH remained normal or low (0.93 and 0.66 μIU/mL) rather than rising as expected. This suggests suppression of the hypothalamic-pituitary axis. The critical difference was in T4 levels. NTIS-1 maintained normal FT4 (13.34 pmol/L) and total T4 (78.41 nmol/L), representing isolated T3 reduction. In contrast, NTIS-2 had low FT4 (9.97 pmol/L) and low total T4 (52.75 nmol/L), consistent with “combined low T3 and T4”.

**Figure 2:**
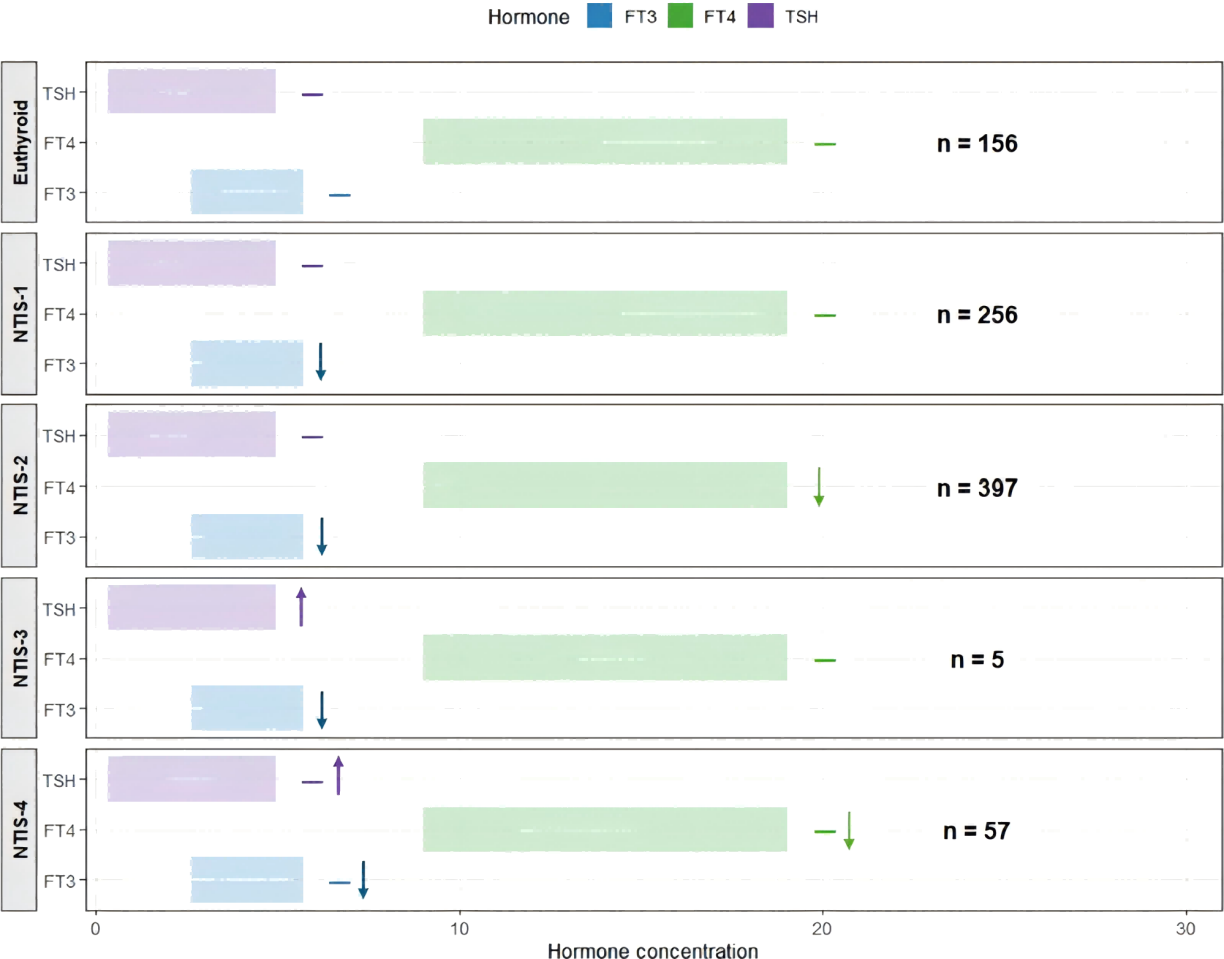
Thyroid hormone levels across thyroid status groups. Horizontal bars show median levels of FT3 (blue), FT4 (green), and TSH (purple) in euthyroid and NTIS subgroups. Arrows indicate direction of change from reference range. Data are shown as median with IQR. FT3, free triiodothyronine; FT4, free thyroxine; TSH, thyroid-stimulating hormone; IQR, interquartile range; NTIS, non-thyroidal illness syndrome.

NTIS-3 showed a different pattern. These patients had low FT3 (2.69 pmol/L) but their TSH rose to 4.38 μIU/mL, near the upper limit of normal. This compensatory response suggests that the hypothalamic-pituitary axis was still functioning. However, this subtype was rare with only 5 patients. NTIS-4 patients had near-normal FT3 (3.58 pmol/L) and total T3 (1.30 nmol/L). They showed only mild reductions in FT4 and total T4, representing a milder form of thyroid dysfunction (**Table 2**, **Figure 2**).

**Table 2:** Thyroid hormones and related markers by thyroid status groups.

Variable	Euthyroid (N = 156)	NTIS-1 (N = 256)	NTIS-2 (N = 397)	NTIS-3 (N = 5)	NTIS-4 (N = 57)	p value
Thyroid hormones						
FT3, pmol/L	3.58 (3.35,3.98)	2.33 (2.00,2.72)	1.81 (1.54,2.18)	2.69 (2.51,2.70)	3.58 (3.23,4.03)	<0.01
FT4, pmol/L	14.16 (12.96,15.32)	13.34 (12.63,14.42)	9.97 (8.73,11.02)	13.71 (13.33,13.94)	11.50 (10.73,12.34)	<0.01
TT3,nmol/L	1.33 (1.15,1.50)	0.81 (0.70, 0.95)	0.66 (0.55, 0.76)	0.85 (0.80, 0.92)	1.30 (1.15, 1.45)	<0.01
TT4, nmol/L	101.16 (85.79,112.92)	78.41 (70.69,90.34)	52.75 (43.02,64.8)	91.14 (83.41,98.28)	79.37 (67.81,91.73)	<0.01
TSH, μIU/mL	1.74 (1.12,2.39)	0.93 (0.40,1.69)	0.66 (0.18,1.38)	4.38 (4.30,4.47)	2.43 (0.98,4.14)	<0.01
Thyroid-related markers						
TPOAb-positive, n (%)	19 (12.3%)	41 (16.0%)	81 (20.4%)	1 (20.0%)	10 (17.5%)	0.233
High thyroglobulin, n (%)	14 (10.4%)	27 (13.1%)	26 (8.2%)	0 (0.0%)	4 (8.3%)	0.396
High rT3, n (%)	26 (19.3%)	81 (39.5%)	118 (37.3%)	3 (60.0%)	4 (8.5%)	<0.01
TGab_positive	36 (23.4%)	89 (34.8%)	162 (40.8%)	2 (40.0%)	19 (33.3%)	<0.01

Continuous variables: median (interquartile range); categorical variables: No. (%). Comparisons used Kruskal-Wallis tests (continuous) and chi-square tests (categorical). FT3, free triiodothyronine; FT4, free thyroxine; TT3, total triiodothyronine; TT4, total thyroxine; TSH, thyroid-stimulating hormone; TPOAb, thyroid peroxidase antibody; TGAb, thyroglobulin antibody; rT3, reverse triiodothyronine.

### Thyroid-related markers

High reverse T3 became progressively more common in NTIS subtypes. Only 19.3% of euthyroid patients had elevated rT3, compared to 39.5% in NTIS-1, 37.3% in NTIS-2, and 60.0% in NTIS-3 (p<0.01). Interestingly, NTIS-4 had the lowest rate at 8.5%, suggesting less severe thyroid axis disruption.

**Figure 3:**
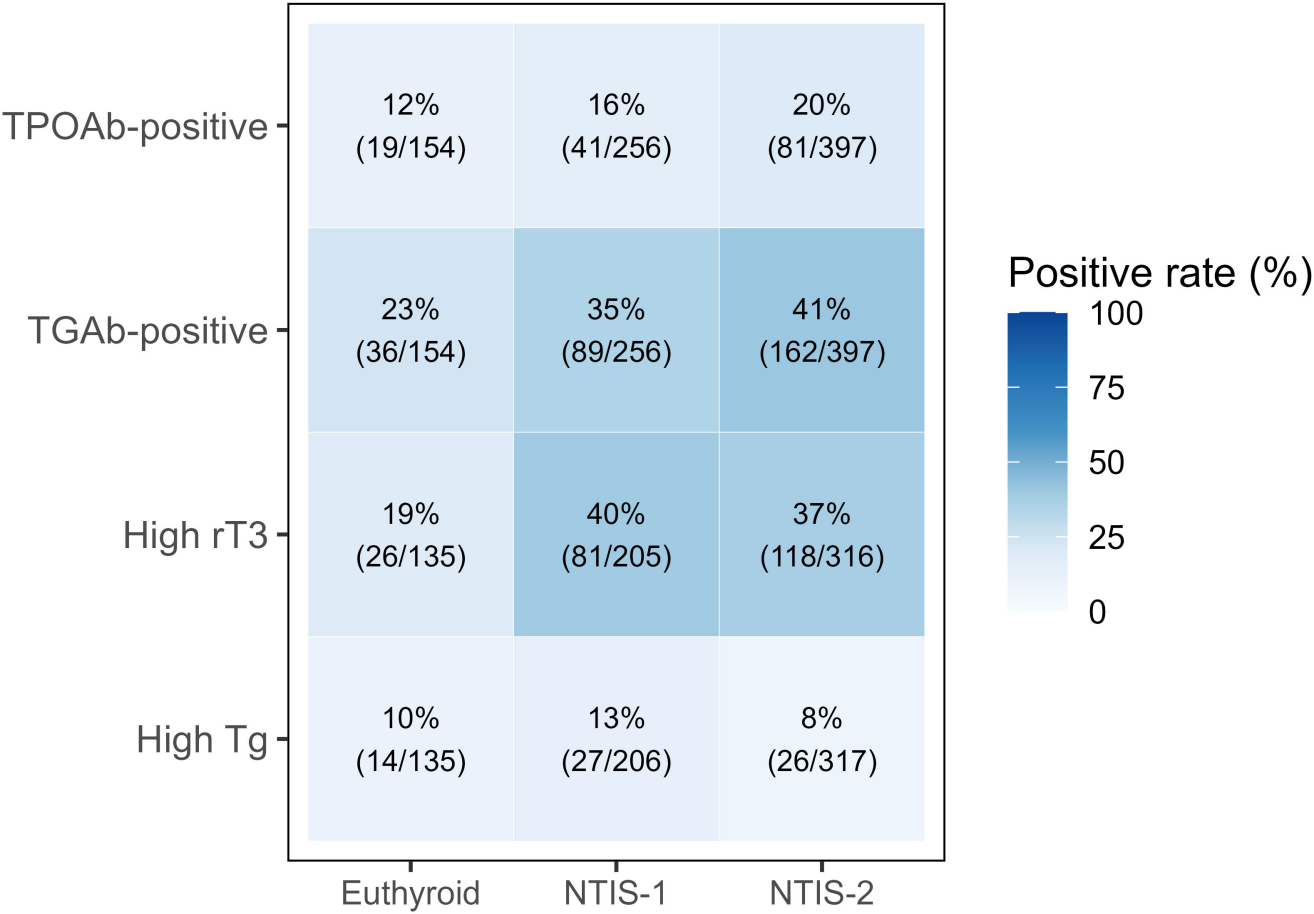
Prevalence of thyroid-related markers by thyroid status. Heatmap shows positive rates (%) for four thyroid-related markers across euthyroid, NTIS-1, and NTIS-2 groups. Color intensity indicates percentage of positive cases. Numbers show counts (positive/total). TPOAb, thyroid peroxidase antibody; TGAb, thyroglobulin antibody; rT3, reverse triiodothyronine; Tg, thyroglobulin; NTIS, non-thyroidal illness syndrome.

Thyroid antibody positivity also increased with NTIS. TGAb-positive rates rose progressively from 23.4% in euthyroid patients to 34.8% in NTIS-1 and 40.8% in NTIS-2 (p<0.01). TPOAb-positive showed a similar increasing trend from 12.3% in euthyroid to 20.4% in NTIS-2, but this did not reach statistical significance (p=0.233). Elevated thyroglobulin was uncommon across all groups, ranging from 0% to 13.1% with no significant differences (p=0.396) (**Table 2**). Due to small sample sizes, marker distribution was visualized only for euthyroid, NTIS-1, and NTIS-2 groups (**Figure 3**).

### NTIS and in-hospital mortality

Crude mortality was higher in patients with NTIS compared with euthyroid patients (82.7% vs 69.7%, p < 0.01). After multivariable adjustment, NTIS remained independently associated with increased in-hospital mortality, with an adjusted odds ratio (OR) of 3.14 (95% confidence interval [CI] 1.52–7.00, p = 0.003).

**Figure 4:**
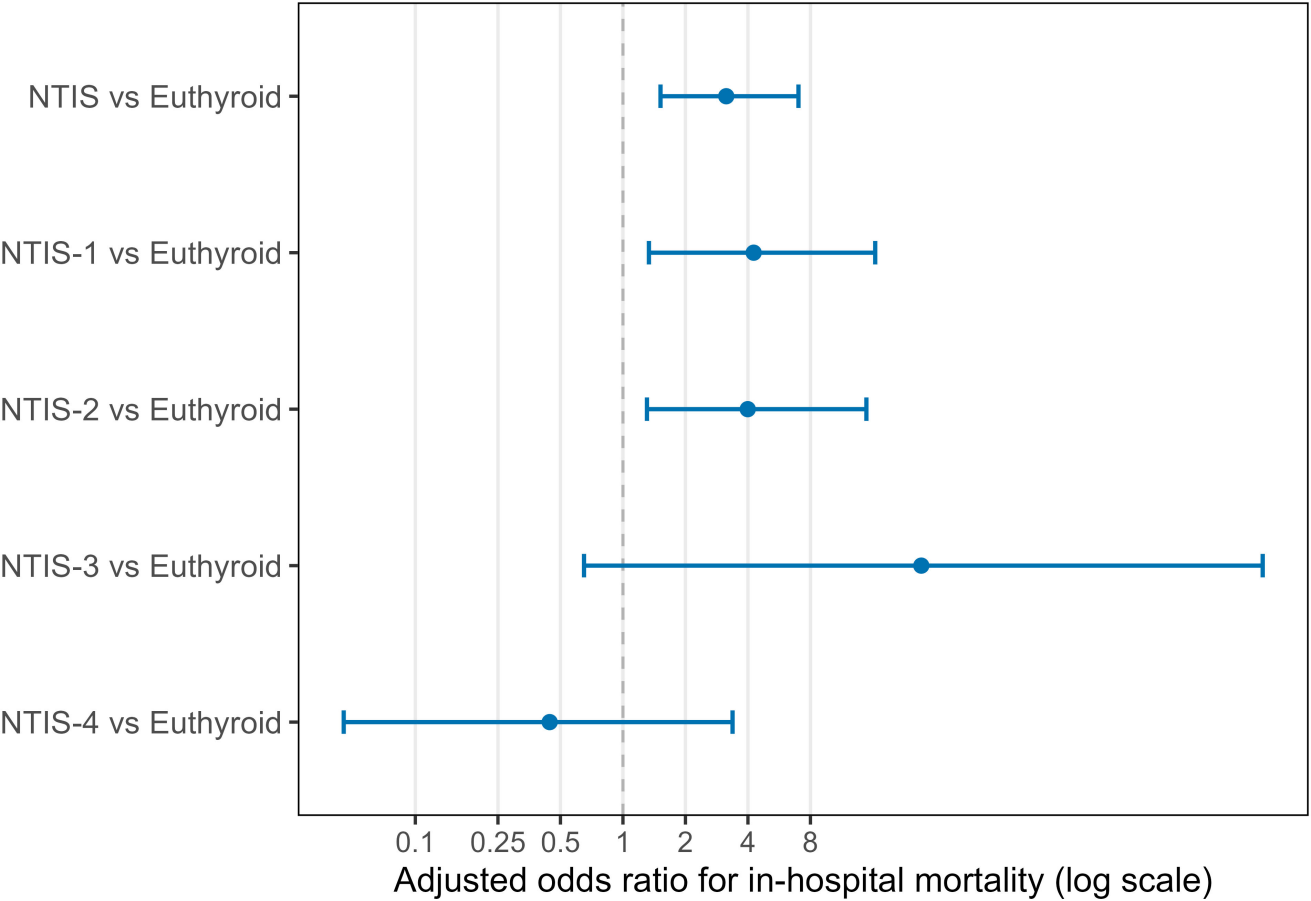
Association between NTIS and in-hospital mortality. Forest plot shows adjusted odds ratios for in-hospital mortality comparing NTIS overall and each subtype to euthyroid patients. Points show odds ratios with 95% confidence intervals. Estimates from multivariable logistic regression models. NTIS, non-thyroidal illness syndrome.

When individual NTIS subtypes were examined, both NTIS-1 and NTIS-2 were significantly associated with higher mortality risk. NTIS-1 was associated with a 4.26-fold increase in adjusted odds of death (95% CI 1.33–16.41, p = 0.022), and NTIS-2 with a 3.99-fold increase (95% CI 1.31–14.88, p = 0.024). NTIS-3 demonstrated a markedly elevated point estimate (adjusted OR 27.35); however, the confidence interval was extremely wide (95% CI 0.65–1206.25) and the association did not reach statistical significance (p = 0.068), reflecting substantial statistical uncertainty likely attributable to the very small sample size of this subgroup (n = 5). NTIS-4 was not significantly associated with mortality (adjusted OR 0.44, 95% CI 0.05–3.37, p = 0.447) (**Table 3**, **Figure 4**).

**Table 3:** Association between NTIS and in-hospital mortality.

Comparison	Adjusted OR (95% CI)	p value
NTIS vs Euthyroid	3.14 (1.52–7.00)	0.003
NTIS-1 vs Euthyroid	4.26 (1.33–16.41)	0.022
NTIS-2 vs Euthyroid	3.99 (1.31–14.88)	0.024
NTIS-3 vs Euthyroid	27.35 (0.65–1206.25)	0.068
NTIS-4 vs Euthyroid	0.44 (0.05–3.37)	0.447

Odds ratios and 95% CIs from multivariable logistic regression adjusted for age, sex, comorbidity index, SOFA score, infection site, and key laboratory values. Reference group is euthyroid patients. OR, odds ratio; CI, confidence interval.

### Thyroid markers and clinical outcomes

**Figure 5:**
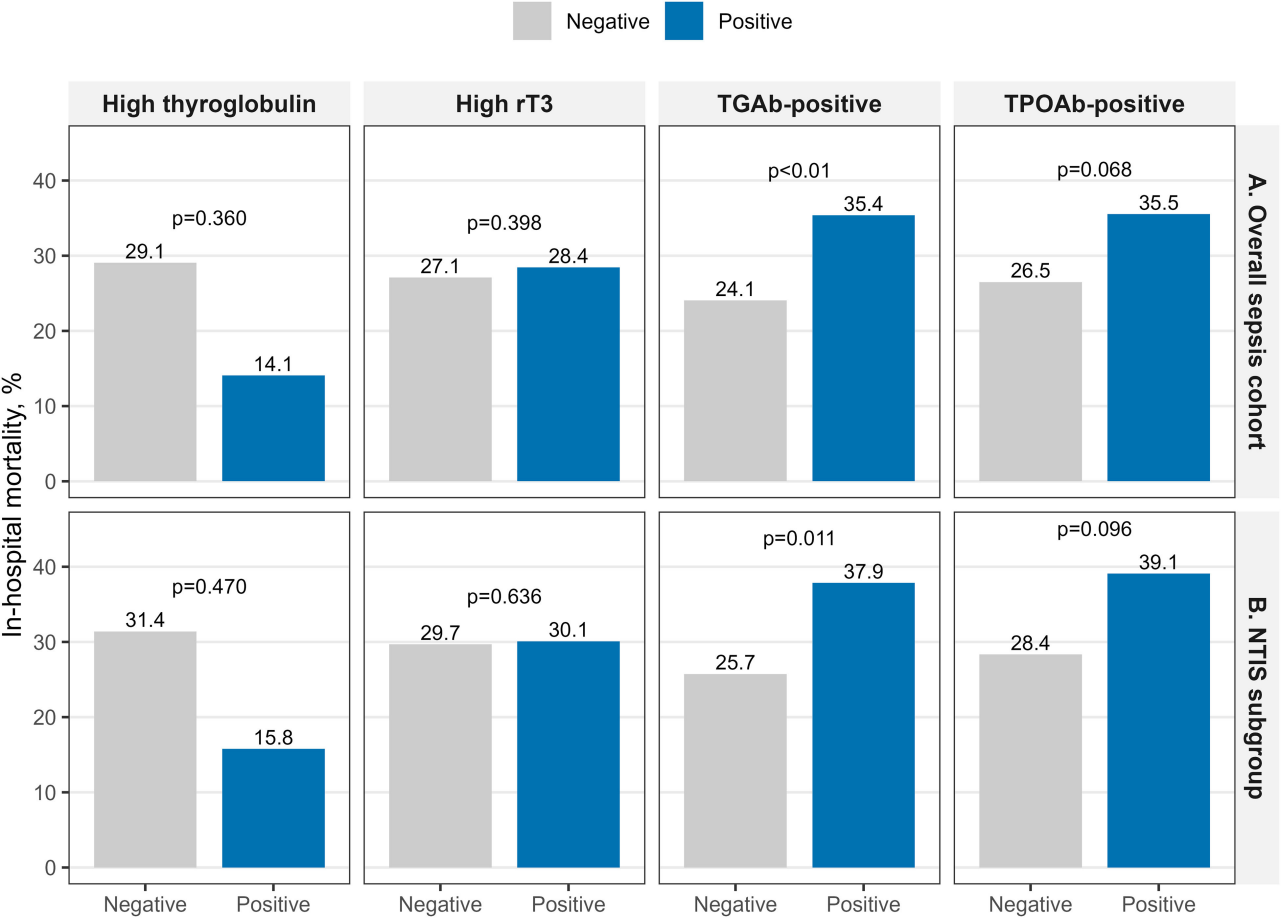
In-hospital mortality by thyroid marker status in sepsis subgroups. Bar charts compare in-hospital mortality (%) between marker-negative (gray) and marker-positive (blue) patients for four thyroid markers. Data are shown separately for two sepsis cohorts **(A)** overall sepsis cohort; **(B)** NTIS subgroup. P values from chi-square tests. TPOAb, thyroid peroxidase antibody; TGAb, thyroglobulin antibody; rT3, reverse triiodothyronine; Tg, thyroglobulin.

TGAb positivity emerged as the strongest predictor of mortality. In all sepsis patients, TGAb-positive patients had higher crude mortality (35.4% vs 24.1%) and 2.15 times higher adjusted odds of death (95% CI 1.30-3.60, p<0.01). This association remained significant in NTIS patients only, with an adjusted OR of 1.97 (95% CI 1.17-3.33, p=0.011). TPOAb positivity showed a similar trend with higher mortality (35.5% vs 26.5%) and an adjusted OR of 1.74, but this did not quite reach statistical significance (95% CI 0.96-3.14, p=0.068). Neither elevated rT3 nor high thyroglobulin predicted mortality in adjusted analyses (**Table 4**, **Figure 5**).

**Table 4:** Association between thyroid markers and in-hospital mortality.

Marker	Patients number	Marker-negative	Marker-positive	Crude OR (95% CI)	Adjusted OR (95% CI)	p value
Overall Sepsis Cohort						
TPOAb-positive	869	190/717 (26.5%)	54/152 (35.5%)	1.53 (1.05–2.21)	1.74 (0.96–3.14)	0.068
TGAb-positive	869	135/561 (24.1%)	109/308 (35.4%)	1.73 (1.28–2.34)	2.15 (1.30–3.60)	<0.01
High rT3	708	129/476 (27.1%)	66/232 (28.4%)	1.07 (0.75–1.51)	1.31 (0.70–2.42)	0.398
High thyroglobulin	711	186/640 (29.1%)	10/71 (14.1%)	0.40 (0.19–0.76)	0.61 (0.20–1.67)	0.360
NTIS Subgroup						
TPOAb-positive	715	165/58x2 (28.4%)	52/133 (39.1%)	1.62 (1.09–2.40)	1.68 (0.91–3.08)	0.096
TGAb-positive	715	114/443 (25.7%)	103/272 (37.9%)	1.76 (1.27–2.43)	1.97 (1.17–3.33)	0.011
High rT3	573	109/367 (29.7%)	62/206 (30.1%)	1.02 (0.70–1.48)	1.17 (0.61–2.24)	0.636
High thyroglobulin	576	163/519 (31.4%)	9/57 (15.8%)	0.41 (0.18–0.82)	0.66 (0.20–1.96)	0.470

Mortality shown as deaths/total (%). Crude and adjusted odds ratios with 95% CIs from logistic regression models. Adjusted models controlled for age, sex, comorbidity index, SOFA score, infection site, and key laboratory values.

**Figure 6:**
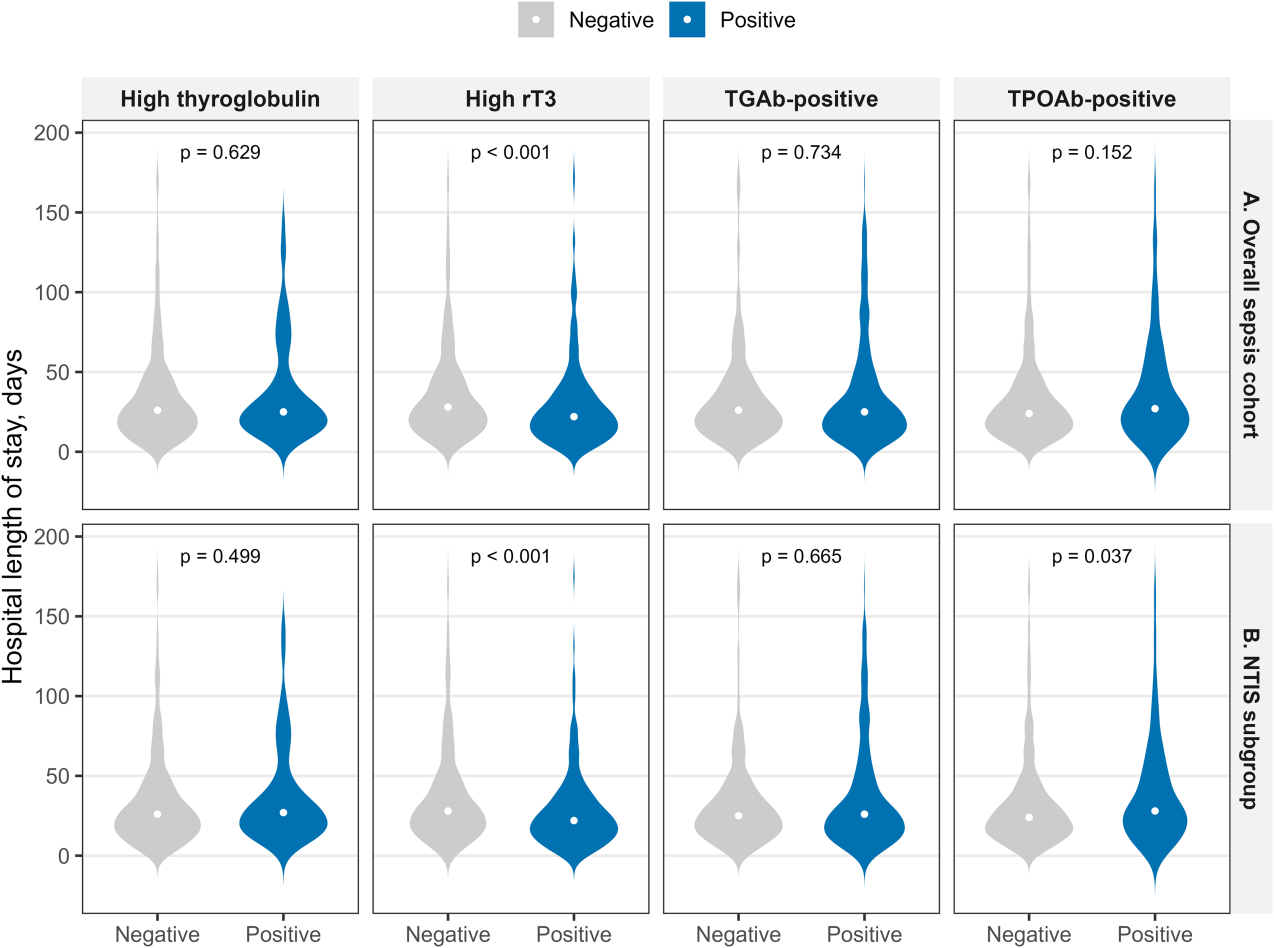
Hospital length of stay by thyroid marker status in sepsis subgroups. Violin plots show hospital length of stay (days) for marker-negative (gray) and marker-positive (blue) patients across four thyroid markers. Data are shown separately for two sepsis cohorts **(A)** overall sepsis cohort; **(B)** NTIS subgroup. White dots show median values. P values from Mann-Whitney test. TPOAb, thyroid peroxidase antibody; TGAb, thyroglobulin antibody; rT3, reverse triiodothyronine; Tg, thyroglobulin.

Hospital length of stay showed different patterns. Patients with elevated rT3 had significantly shorter stays in both the overall cohort (22 vs 28 days, p<0.01) and in NTIS patients (22 vs 28 days, p<0.01). However, this may reflect early mortality rather than faster recovery, given that rT3 did not predict survival benefit. In contrast, TPOAb positivity was associated with longer hospital stay only in NTIS patients (28 vs 24 days, p=0.037), possibly indicating a more prolonged illness course in those with thyroid autoimmunity. TGAb status and thyroglobulin levels did not significantly affect length of stay (**Table 5**, **Figure 6**).

**Table 5:** Hospital length of stay by thyroid marker status.

Marker	Patients,No.	Marker negative, LOS days	Marker positive, LOS days	p value
Overall Sepsis Cohort				
TPOAb-positive	860	24 (15, 41)	27 (16, 51)	0.152
TGab-positive	860	26 (15, 41)	25 (14, 45)	0.734
High rT3	703	28 (17, 46)	22 (13, 36)	<0.01
High Tg	706	26 (15, 43)	25 (16, 43)	0.629
NTIS Subgroup				
TPOAb-positive	707	24 (15, 39)	28 (17, 52)	0.037
TGab-positive	707	25 (15, 40)	26 (14, 46)	0.665
High rT3	568	28 (17, 46)	22 (13, 36)	<0.01
High Tg	571	26 (15, 42)	27 (16, 42)	0.499

Length of stay shown as median (interquartile range) in days. Comparisons used Mann-Whitney tests. LOS, length of stay; Tg, thyroglobulin.

## Discussion

In this cohort of 871 patients with sepsis, non-thyroidal illness syndrome (NTIS) was highly prevalent, affecting 82% of patients who underwent thyroid function testing. This prevalence is comparable to that reported in previous intensive care unit (ICU) studies (5, 6, 8). Consistent with earlier observations, patients with NTIS were older, carried more comorbidities, and had higher SOFA scores than euthyroid patients (9, 15). These findings reinforce the link between NTIS and critical illness severity (9, 15).

We identified four NTIS subtypes based on thyroid hormone profiles. NTIS-1 was defined by an isolated reduction in triiodothyronine (T3). NTIS-2 was characterized by a concurrent reduction in T3 and thyroxine (T4). In NTIS-3, the decrease in T3 was accompanied by a preserved or compensatory response of thyroid-stimulating hormone (TSH), whereas NTIS-4 was characterized by largely preserved hormone levels with only mild abnormalities, reflecting a more heterogeneous pattern within the NTIS spectrum.In adjusted analyses accounting for disease severity and other potential confounders, NTIS remained independently associated with in-hospital mortality, with the odds of death increased by more than threefold. Similar associations have been reported in earlier meta-analyses involving septic populations (8, 14, 16).

Mortality risk was highest in the NTIS-1 and NTIS-2 subtypes, both of which were associated with an approximately fourfold increase in risk, consistent with the findings reported by Padhi et al. (16). This pattern suggests a potential role of T3 deficiency. Experimental studies have shown that reduced availability of T3 may impair mitochondrial function and tissue repair, thereby contributing to organ dysfunction (10, 27).

NTIS was associated with several markers of disease severity. In both unadjusted and adjusted analyses, NTIS was associated with higher mortality. Although NTIS patients had greater baseline disease severity, the association persisted after adjustment for major confounders, suggesting that NTIS may represent more than a simple marker of illness severity.One possible interpretation is that NTIS reflects an endocrine response that is not entirely adaptive in the setting of critical illness. Prior reviews have described a biphasic pattern of thyroid hormone changes, in which early suppression may be energy conserving, while persistent suppression may become detrimental (12, 25, 26). Although low T3 levels have been proposed as protective in some settings (11, 28), other studies have linked sustained reductions to impaired recovery and organ dysfunction (13, 25). In our cohort, the observed associations were more consistent with the latter interpretation and with reports relating persistent NTIS to prolonged organ dysfunction (15, 30).

Among thyroid-related biomarkers, thyroglobulin antibody (TGAb) positivity emerged as the strongest predictor of mortality, doubling the risk of death in both the overall sepsis cohort and the NTIS subgroup. This observation is concordant with prior reports associating thyroid autoantibodies with unfavorable ICU outcomes (22, 24). Thyroid autoimmunity may reflect an underlying immune dysregulation that compromises host defense, alters cytokine signaling, and exacerbates inflammatory responses during sepsis (18, 21). Thyroid peroxidase antibody (TPOAb) positivity showed a similar trend toward increased mortality but did not reach statistical significance, possibly due to lower prevalence or biological differences between antibody types. Elevated reverse T3 (rT3) levels were associated with shorter hospital stays, a finding that likely reflects early mortality rather than accelerated recovery, as rT3 was not associated with improved survival. Consistent with this interpretation, rT3 has been described as a marker of metabolic suppression in critical illness (28). In contrast, TPOAb positivity was associated with prolonged hospitalization among NTIS patients, suggesting a more protracted disease course in the presence of thyroid autoimmunity.

From a clinical perspective, these findings suggest that thyroid function assessment may enhance risk stratification in sepsis. Identification of NTIS-1 or NTIS-2 patterns could help recognize patients at particularly high risk of death (5, 6, 14), while TGAb measurement may provide prognostic information beyond established severity scores. The high prevalence of thyroid autoantibodies among patients with NTIS also raises the possibility that autoimmunity contributes to susceptibility to severe illness. The role of thyroid hormone replacement therapy in this context remains controversial. Randomized trials and meta-analyses have generally failed to demonstrate a survival benefit and have even suggested potential harm in unselected ICU populations (29, 30). Although our study does not address therapeutic interventions, the observed heterogeneity across NTIS subtypes and immune profiles suggests that treatment effects may differ among patient subgroups. Future interventional studies should therefore consider stratifying participants by NTIS subtype and thyroid antibody status.

This study has several strengths, including a large sample size, comprehensive assessment of thyroid hormones and related biomarkers, and extensive adjustment for clinical confounders. However, several limitations merit consideration. The single-center, retrospective design may limit generalizability. Thyroid function was measured only once at the time of sepsis diagnosis, and serial measurements might better capture dynamic changes in thyroid status. The small number of patients with NTIS-3 precludes firm conclusions regarding this subtype. In addition, we lacked data on thyroid hormone therapy during hospitalization and on long-term outcomes after discharge. Although thyroid-specific therapy is uncommon in acute sepsis settings, the absence of such information may have influenced hormone and antibody measurements and therefore could not be fully accounted for in our analyses. As with all observational studies, causality cannot be inferred.

In conclusion, NTIS is highly prevalent in patients with sepsis and independently predicts mortality beyond established measures of illness severity. NTIS-1 and NTIS-2 subtypes are associated with the highest risk of death, and TGAb positivity represents a strong independent prognostic marker. Together, these findings indicate that thyroid function testing, including antibody assessment, may improve risk stratification in sepsis. Further research is warranted to determine whether NTIS subtyping or thyroid autoimmunity can inform personalized therapeutic strategies.

## Conclusion

In this cohort of 871 patients with sepsis, non-thyroidal illness syndrome (NTIS) was highly prevalent and independently associated with increased mortality after adjustment for illness severity. Among the identified subtypes, NTIS-1 and NTIS-2 were associated with the highest risk of death. In addition, thyroglobulin antibody (TGAb) positivity emerged as a strong and independent predictor of mortality in both the overall cohort and the NTIS subgroup. Collectively, these findings indicate that assessment of thyroid function, particularly when combined with thyroid antibody measurement, may enhance risk stratification in patients with sepsis. Future studies are warranted to determine whether NTIS subtyping or thyroid antibody status can be used to guide individualized therapeutic strategies.

## Data Availability

The raw data supporting the conclusions of this article will be made available by the authors, without undue reservation.

## References

[1] Singer M Deutschman CS Seymour CW Shankar-Hari M Annane D Bauer M . The third international consensus definitions for sepsis and septic shock (Sepsis-3). JAMA. (2016) 315:801–10. doi: 10.1001/jama.2016.0287, PMID: 26903338 PMC4968574

[2] Rudd KE Johnson SC Agesa KM Shackelford KA Tsoi D Kievlan DR . Global, regional, and national sepsis incidence and mortality, 1990–2017: analysis for the Global Burden of Disease Study. Lancet. (2020) 395:200–11. doi: 10.1016/S0140-6736(19)32989-7, PMID: 31954465 PMC6970225

[3] Kaukonen KM Bailey M Pilcher D Cooper DJ Bellomo R . Mortality related to severe sepsis and septic shock among critically ill patients in Australia and New Zealand, 2000–2012. JAMA. (2014) 311:1308–16. doi: 10.1001/jama.2014.2637, PMID: 24638143

[4] van der Poll T van de Veerdonk FL Scicluna BP Netea MG . The immunopathology of sepsis and potential therapeutic targets. Nat Rev Immunol. (2017) 17:407–20. doi: 10.1038/nri.2017.36, PMID: 28436424

[5] Fliers E Bianco AC Langouche L Boelen A . Thyroid function during critical illness. Lancet Diabetes Endocrinol. (2015) 3:816–25. doi: 10.1016/S2213-8587(15)00225-9, PMID: 26071885 PMC4979220

[6] Vidart J Jaskulski P Kunzler ALF de Souza DS Vargas KV da Silva Jaques M . Non-thyroidal illness syndrome predicts outcome in adult critically ill patients: a systematic review and meta-analysis. Endocr Connect. (2022) 11:e210504. doi: 10.1530/EC-21-0504, PMID: 35015701 PMC8859965

[7] Krug B Bercker S Reimann D Stumvoll M Schroeder C . Non-thyroidal illness syndrome is no independent predictor for mortality in ICU patients. BMC Anesthesiol. (2023) 23:119. doi: 10.1186/s12871-023-02015-1, PMID: 37003983 PMC10064728

[8] Wironegoro R Kloping NA Witarto AP Putra ICS Pradigda FF Nugraha RA . Prognostic significance of non-thyroidal illness syndrome in sepsis and septic shock: a systematic review and meta-analysis. Anaesth Pain Intensive Care. (2022) 26:54–62. doi: 10.35975/apic.v26i1.1768

[9] Geetha NK Vaishnavi KISN Jagadeswar K Shankar AG . Prognostic significance of non-thyroidal illness syndrome in sepsis. Rom J Infect Dis. (2024) 27:175–81. doi: 10.37897/rjid.2024.2.9

[10] Fontes KN Cabanelas A Bloise F de Souza LL Pelosi P de Castro Faria Neto HC . Differential regulation of thyroid hormone metabolism target genes during non-thyroidal illness syndrome triggered by fasting or sepsis in adult mice. Front Physiol. (2017) 8:828. doi: 10.3389/fphys.2017.00828, PMID: 29118715 PMC5661015

[11] Warner MH Beckett GJ . Mechanisms behind the non-thyroidal illness syndrome: an update. J Endocrinol. (2010) 205:1–13. doi: 10.1677/JOE-09-0412, PMID: 20016054

[12] Chatzitomaris A Hoermann R Midgley JEM Hering S Urban A Dietrich B . Thyroid allostasis—adaptive responses of thyrotropic feedback control to conditions of strain and stress. Front Endocrinol (Lausanne). (2017) 8:163. doi: 10.3389/fendo.2017.00163, PMID: 28775711 PMC5517413

[13] Langouche L Van den Berghe G . Thyroid function and therapy during critical illness. Nat Rev Endocrinol. (2014) 10:731–42. doi: 10.1038/nrendo.2014.135, PMID: 41872693

[14] Wang F Pan W Wang H Wang S Pan S Ge J . Relationship between thyroid function and ICU mortality: a prospective observational study. Crit Care. (2012) 16:R11. doi: 10.1186/cc11145, PMID: 22257427 PMC3396242

[15] Foks M Dudek A Polok K Gastol P Nowak-Kozka I Zukowski M . Non-thyroidal illness syndrome in sepsis and its association with clinical outcome: a prospective observational study. Anaesthesiol Intensive Ther. (2019) 51:202–9.

[16] Padhi R Kabi S Panda B Jagati S . Prognostic significance of non-thyroidal illness syndrome in critically ill adult patients with sepsis. Int J Crit Illn Inj Sci. (2018) 8:165–72. 10.4103/IJCIIS.IJCIIS_29_17 PMC6116306 30181975

[17] Afsana F Fatema K Ahsan A Bhowmik B Siddique T . Outcome of critically ill patients with non-thyroidal illness. BIRDEM Med J. (2020) 11:47–51. doi: 10.3329/birdem.v11i1.51029, PMID: 40208441

[18] Boelen A Fliers E . The role of the immune system in the pathophysiology of non-thyroidal illness syndrome. Endocr Rev. (2021) 42:123–32.

[19] Fliers E Boelen A . An update on non-thyroidal illness syndrome. J Endocrinol Invest. (2021) 44:1597–607. doi: 10.1007/s40618-020-01482-4, PMID: 33320308 PMC8285315

[20] Hollowell JG Staehling NW Flanders WD Hannon WH Gunter EW Spencer CA . T4, and thyroid antibodies in the United States population (1988 to 1994): National Health and Nutrition Examination Survey (NHANES III). J Clin Endocrinol Metab. (2002) 87:489–99. doi: 10.1210/jcem.87.2.8182, PMID: 11836274

[21] Antonelli A Ferrari SM Ragusa F Elia G Paparo SR Ruffilli I . The interplay between thyroid autoimmunity and infection. Endocrine. (2022) 76:247–57.

[22] Antonelli A Ferrari SM Corrado A Di Domenicantonio A Fallahi P . Autoimmunity and thyroid cancer: review of the literature. Eur J Endocrinol. (2019) 180:R261–77.

[23] Chen Y Sjölinder M Wang X Altenbacher G Bergstrand M Wai SN . Thyroid hormone enhances nitric oxide-mediated bacterial clearance and promotes survival after meningococcal infection. PLoS One. (2012) 7:e41445. doi: 10.1371/journal.pone.0041445, PMID: 22844479 PMC3402396

[24] Schwarz Y Percik R Oberman B Yaffe D Zimlichman E Tirosh A . Sick euthyroid syndrome on presentation of patients with COVID-19: a potential marker for disease severity. Endocr Pract. (2021) 27:101–9. doi: 10.1016/j.eprac.2021.01.001, PMID: 33551316 PMC7834506

[25] Fliers E Boelen A Wiersinga WM . Mechanisms and clinical implications of non-thyroidal illness syndrome. Nat Rev Endocrinol. (2015) 11:406–18. doi: 10.1038/nrendo.2015.39, PMID: 25752275

[26] Langouche L Jacobs A Van den Berghe G . Nonthyroidal illness syndrome across the ages. J Endocr Soc. (2019) 3:2313–25. 10.1210/js.2019-00325 PMC6853682 31745528

[27] Carreras L Riaño I Vivanco A Rey C Diaz JJ Concha-Torre A . Non-thyroidal illness syndrome and its relationship with mortality risk in critically ill children. Front Pediatr. (2023) 11:1142332. doi: 10.3389/fped.2023.1142332, PMID: 36937966 PMC10020518

[28] Economidou F Douka E Tzanela M Nanas S Kotanidou A . Thyroid function during critical illness. Hormones (Athens). (2011) 10:117–24. doi: 10.14310/horm.2002.1309, PMID: 21724536

[29] Van den Berghe G de Zegher F Baxter RC Veldhuis JD Wouters P Bowers CY . Neuroendocrine and metabolic effects of growth hormone secretagogues in prolonged critical illness. J Clin Endocrinol Metab. (2001) 86:1573–81. doi: 10.1210/jcem.86.4.7399, PMID: 11297589

[30] Bello G Pennisi MA Montini L Silva S Maviglia R Cavallaro F . Nonthyroidal illness syndrome and prolonged mechanical ventilation in patients admitted to the ICU. Chest. (2009) 135:1448–54. doi: 10.1378/chest.08-1816, PMID: 19255297

