# Supplementary material for: Non-thyroidal illness syndrome subtypes and mortality in sepsis: associations with thyroid autoantibodies
Source: Front Endocrinol (Lausanne). 2026 Mar 27;17:1767422. doi: 10.3389/fendo.2026.1767422 (PMC13065710; doi:10.3389/fendo.2026.1767422)
Supplement: Supplementary file 1 [file Table1.pdf]

Supplementary Table S1. Baseline Characteristics of Patients With and Without Thyroid Function Testing

Variable	Thyroid panel (N=961)	No thyroid labs (N=899)	p value
**Demographics**			
Age, years			<0.001
18-39	179 (18.6)	118 (13.0)	
40-64	393 (41.5)	329 (36.6)	
≥65	377 (39.2)	464 (51.6)	
Male sex	591 (61.5)	603 (67.1)	0.087
**Vital Signs**			
Heart rate	122.0 (104.0-139.0)	120.0 (100.0-140.0)	0.029
MAP, mm Hg	78.0 (70.0-88.0)	79.0 (70.0-89.0)	0.846
Temperature, °C	38.9 (38.2-39.8)	38.9 (38.2-39.6)	0.415
GCS score	15.0 (15.0-15.0)	15.0 (15.0-15.0)	0.495
**Comorbidities and Risk Factors**			
Diabetes mellitus	167 (17.3)	133 (14.8)	0.071
History of DVT	343 (35.7)	312 (34.7)	0.392
Current smoking	235 (24.5)	168 (18.7)	<0.001
Alcohol use	174 (18.1)	120 (13.3)	0.002
**Infection site**			**<0.001**
Respiratory	364 (37.9)	254 (28.3)	
Abdominal	256 (26.6)	327 (36.4)	
Urinary	20 (2.1)	18 (2.0)	
Bloodstream	10 (1.0)	10 (1.1)	
Central nervous system	7 (0.7)	5 (0.6)	
Skin/soft tissue	13 (1.4)	43 (4.8)	
Unknown	247 (25.7)	227 (25.2)	
**Severity**			
APS score	8.0 (5.0-10.0)	8.0 (5.0-11.0)	0.592
APACHE II score	11.0 (4.0-20.0)	13.0 (4.0-21.0)	0.139
SIRS score	3.0 (2.0-3.0)	3.0 (2.0-3.0)	0.523
qSOFA score	1.0 (0.0-1.0)	1.0 (0.0-1.0)	0.324
SOFA score	4.0 (1.0-7.0)	4.0 (2.0-7.0)	0.022
Septic shock	93 (9.7)	83 (9.2)	0.612
**Laboratory Values**			
White blood cell count, $\times 10^9$/L	9.1 (5.7-13.6)	10.0 (5.9-14.5)	0.020

Neutrophil count, $\times 10^9$/L	7.3 (4.1-11.4)	8.2 (4.3-12.5)	0.031
Hemoglobin, g/L	99.4 (79.0-117.0)	100.0 (79.0-120.0)	0.436
Hematocrit, %	0.30 (0.24-0.35)	0.30 (0.24-0.35)	0.563
Platelet count, $\times 10^9$/L	147.0 (76.0-235.0)	157.0 (80.0-244.0)	0.317
PT, sec	13.9 (12.7-15.8)	14.1 (12.9-16.0)	0.031
APTT, sec	33.3 (29.6-39.5)	34.3 (30.1-41.8)	0.008
D-dimer	4.0 (1.7-8.2)	4.5 (1.9-8.2)	0.449
Fibrinogen, g/L	3.8 (2.5-5.1)	3.6 (2.4-4.9)	0.110
ALT, U/L	25.0 (14.0-52.0)	22.0 (14.0-48.0)	0.266
AST, U/L	33.0 (20.0-62.0)	32.0 (20.0-63.0)	0.884
Total bilirubin, µmol/L	18.0 (11.7-31.6)	19.4 (12.2-37.3)	0.089
Creatinine, µmol/L	75.0 (55.0-123.0)	75.0 (55.0-120.0)	0.851
Sodium, mmol/L	138.0 (135.0-143.0)	138.0 (135.0-142.0)	0.303
Potassium, mmol/L	3.9 (3.5-4.2)	3.8 (3.5-4.3)	0.923
Lactate, mmol/L	2.0 (1.4-2.9)	2.5 (1.6-4.0)	<0.001
Arterial pH	7.42 (7.38-7.45)	7.41 (7.36-7.45)	0.049
**Outcomes**			
In-hospital mortality	128 (13.3)	154 (17.1)	0.014
Length of stay, days	25.0 (15.0-44.0)	21.0 (10.0-40.0)	<0.001

Continuous variables are presented as median (interquartile range); categorical variables as No. (%). Comparisons used Wilcoxon rank-sum test for continuous variables and chi-square/Fisher's exact test for categorical variables.

Abbreviations: MAP, mean arterial pressure; GCS, Glasgow Coma Scale; DVT, deep vein thrombosis; APS, Acute Physiology Score; APACHE II, Acute Physiology and Chronic Health Evaluation II; SIRS, Systemic Inflammatory Response Syndrome; qSOFA, quick Sequential Organ Failure Assessment; SOFA, Sequential Organ Failure Assessment; PT, prothrombin time; APTT, activated partial thromboplastin time; ALT, alanine aminotransferase; AST, aspartate aminotransferase.
